# Functional identification of a rare vascular endothelial growth factor a (*VEGFA*) variant associating with the nonsyndromic cleft lip with/without cleft palate

**DOI:** 10.1080/21655979.2021.1912547

**Published:** 2021-05-05

**Authors:** Bohui Sun, Yangjia Liu, Wenbin Huang, Qian Zhang, Jiuxiang Lin, Weiran Li, Jieni Zhang, Feng Chen

**Affiliations:** a Department of Orthodontics, Peking University School and Hospital of Stomatology, Beijing, China; b Central Laboratory, Peking University School and Hospital of Stomatology, Beijing, China

**Keywords:** *VEGFA*, mutation, HEPM, osteogenesis, palatogenesis, nonsyndromic cleft lip with or without cleft palate

## Abstract

Vascular endothelial growth factor A (VEGFA) is a crucial growth factor, which participates in multiple processes of human growth and development, such as angiogenesis and osteogenesis and is also necessary for development of palate. The purpose of this study was to investigate the effect of a rare *VEGFA* mutation (NM_001025366.2 773 T > C p.Val258Ala) on the cell functions and osteogenesis. Here, we found that the *VEGFA* mutation has adverse effects on the function of human embryonic palatal plate mesenchymal (HEPM) cells, and may affect the development of palate. The *VEGFA* mutation has adverse effects on promoting cell proliferation and migration and inhibiting apoptosis in HEPM and HEK–293 cells. In addition, the mutant *VEGFA* allele has a negative influence on osteogenesis. Taken together, the rare variant of *the VEGFA* gene had an adverse effect on cell functions and osteogenesis, which may impact the development of the palate. And these findings suggested that *VEGFA* mutation (c.773 T > C) may lead to nonsyndromic cleft lip with or without cleft palate and also provide a new insight into the mechanism of *VEGFA* gene in osteogenesis and palatogenesis.

## GRAPHICAL ABSTRACT

**Figure:**
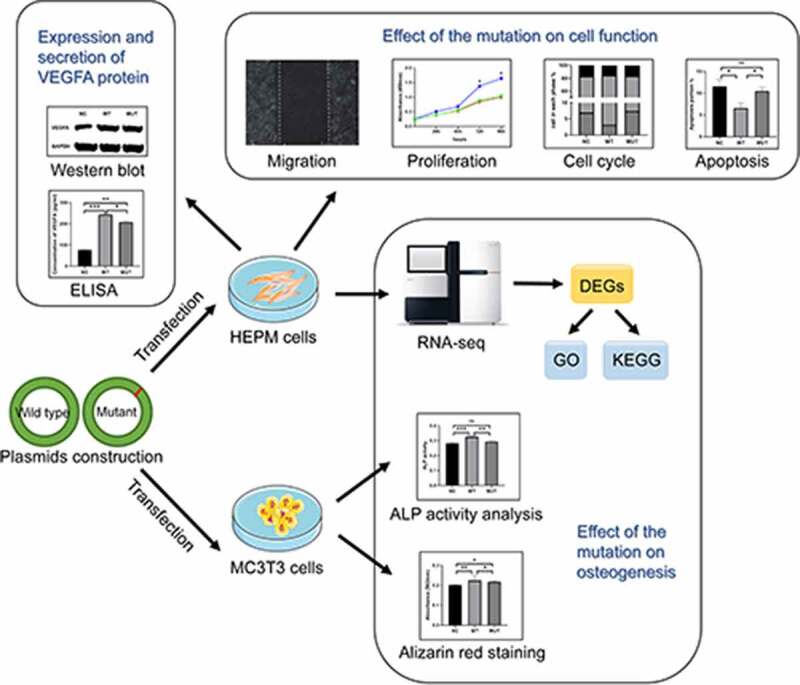

## INTRODUCTION

Vascular endothelial growth factor A (*VEGFA*) is a key factor affecting angiogenesis, which regulates blood vessel formation, development and function. It controls the survival, proliferation and migration of endothelial cells, which further lead to more complicated morphological changes, such as tubular budding, fusion and vascular network formation [1,2]. In addition, *VEGFA* plays a crucial role in many developmental processes, containing neurogenesis and osteogenesis, which are key to organogenesis during embryogenesis [3,4]. *VEGFA* regulates osteogenesis directly or indirectly. On the one hand, *VEGFA* indirectly promoted osteogenesis and osteoblast differentiation by affecting the growth of blood vessels during endochondral and intramembranous osteogenesis [5–7]. Blood vessels provide oxygen, nutrients and growth factors for bone formation. At the same time, angiogenesis is a prerequisite for osteogenesis, because angiogenesis is regarded as the source of bone marrow stromal cells, which are the precursors of bone tissue components. On the other hand, *VEGFA* also directly stimulates non-endothelial cells, such as osteoclasts and osteoblasts, and is thought to be beneficial to the survival of chondrocytes during the formation of long bone [8]. VEGFA receptors are expressed on the membrane of osteocytes, which VEGFA can bind to through autocrine or paracrine, regulating the differentiation and function of osteocytes, and directly participate in the process of bone formation and metabolism [9]. Therefore, hypomorphic mutations in *VEGFA* caused abnormal angiogenesis and osteogenesis, resulting in skeletal defects. Specifically, *VEGFACKO* mice presented a reduction in cell proliferation in palate plate and abnormal palate plate elongation and elevation, eventually, leading to the occurrence of cleft palate. Deficiencies of vascular development and intramembranous ossification were also observed during palatogenesis in the conditional deletion of *VEGFA* (*VEGFA*CKO) mice [10]. Moreover, some studies have also suggested that *VEGFA ^120/120^* mice have craniofacial defects, including cleft palate and developmental disorders in mandibular [11,12].

Cleft lip with or without cleft palate (CL/P) is one of the most common congenital defects of human with the occurrence of 1/700 in in liveborn infant [13]. 70% of CL/P cases have no other congenital or craniofacial structural abnormalities, which are called nonsyndromic cleft lip and/or cleft palate (NSCL/P) [14]. NSCL/P has a complex etiology, which is affected not only by genetic risk factors but also by environmental risk conditions [15]. The application of the genomic tools, such as candidate genes and genome wide association study (GWAS), and animal models provided a more in-depth understanding of the cause of NSCL/P.

In our previous study, we identified a VEGFA mutation (NM_001025366.2 c.773 T > C p.Val258Ala) as a candidate for causing NSCL/P in a hereditary NSCL/P family [16]. However, abnormal palatal development and the mechanism of NSCL/P caused by this VEGFA mutation was still unclear. Here, we explored the functional roles of this mutation in VEGFA and the gene by in vitro experiments. As a result, we further proved that VEGFA gene mutations play an important role in the etiology of NSCL/P.

## MATERIALS AND METHODS

### Cell culture and osteogenic differentiation

The human embryonic palatal mesenchyme (HEPM) cells have been widely applied to the study of palatal growth and development [17,18]. The human embryonic kidney 293 (HEK-293) cells have been often used to study cell function [19]. MC3T3, a preosteoblast cell line, was widely used as a model of osteoblasts [20,21]. HEPM and HEK-293 cells were cultured in Dulbecco’s modified Eagle’s medium (DMEM; Gibco, USA), added with 100 units/mL penicillin, 10% fetal bovine serum (FBS), and 100 μg/mL streptomycin at 37 °C under 5% CO_2_. MC3T3 cells were cultured in α-Modification minimum essential medium (α-MEM; Gibco, USA) under the same conditions. To induce osteoblastic differentiation, MC3T3 cells were cultured in 6-well plates with complete medium added additional osteogenic supplement, including 10 μM dexamethasone, 50 μg/mL VC, and 5 mM β-glycerophosphate.

### Plasmid construction

pVax1-hVEGFA_165_ (addgene, 74466), containing human *VEGFA_165_*, was used as the wild type VEGFA_165_ expression vector (Supplementary Figure 1). Using this plasmid as a template, we have constructed the mutant pVax1-hVEGFA_165_ vector using a point mutation kit (Transgen, China). We designed primers before and after the mutation site, and used PCR technology to replace T at the target site with C. The forward primer sequence is TCAAGCCATCCTGTGCCCCTGATG and the reverse primer sequence is GCACAGGATGGCTTGAAGATGTACT. The mutation vector contained the point mutation in *VEGFA_165_* reported in our previous study: c.773 T > C predicting p.Val258Ala.

**Figure 1.**
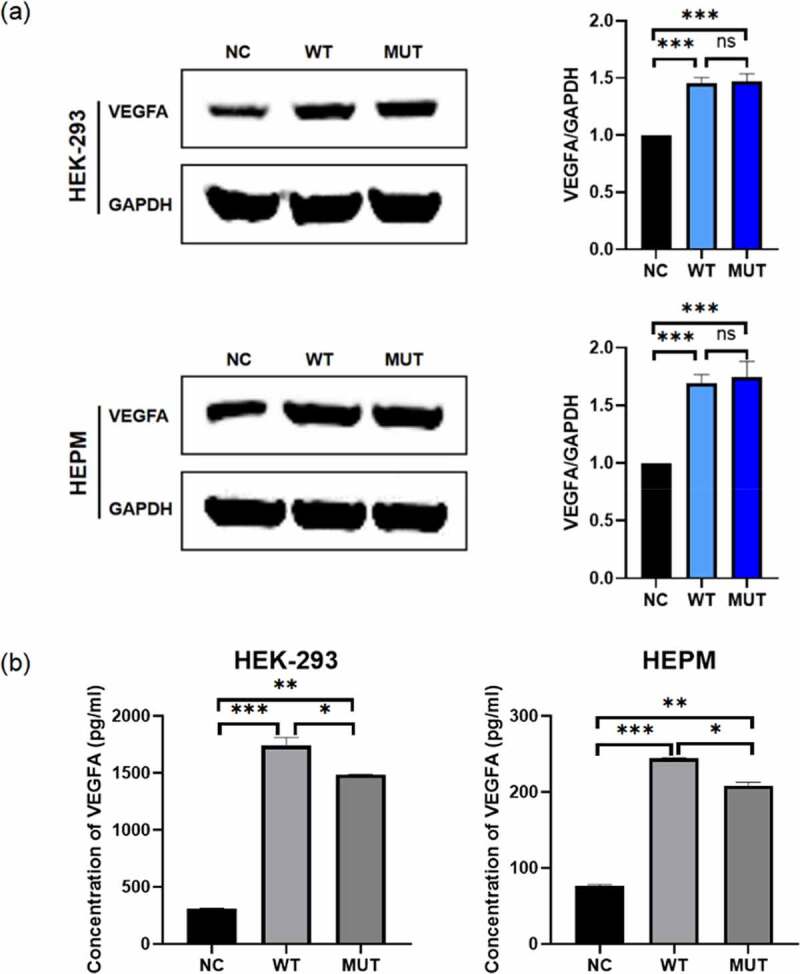
The expression of *VEGFA* in human cell lines. HEPM and HEK-293 cells were transfected to express either the wild-type/mutant *VEGFA* allele or control vector. (a) The VEGFA protein expression levels were estimated by western blot (left); the amounts of VEGFA were determined by densitometry of protein bands (right); GAPDH was the internal control. (b) The VEGFA protein levels in cell supernatants were estimated by ELISA. n = 3 for each group. *P < 0.05, **P < 0.01, ***P < 0.001, no significance

### Transient transfection

HEPM cells, HEK-293 cells and MC3T3 cells were grown in well plates before transient transfection. After reaching approximately 80% confluence at 24 h, wild-type/mutant *VEGFA* plasmids were transfected to cells using FuGENE HD® Reagent (Promega, USA), according to the user’s instruction. Refresh medium 24 hours after transfection. In all experiments involved in this study, the cells transfected with empty vectors were used as the negative control.

### Western blot analysis

HEPM and HEK-293 cells were collected after transfection for 48 hours. RIPA lysis buffer (Cwbiotech, China) was used to extract total proteins and proteins were quantified with a BCA protein quantification kit (Cwbiotech, China). 30 μg total protein samples were separated by SDS-PAGE and transferred to PVDF membranes. Then, protein samples on the PVDF membranes were incubated with a primary anti-VEGFA (Santa Cruz, USA) or anti-GAPDH (Cell Signaling Technology, USA) antibody at 4 °C for 12 hours and then with secondary antibodies at room temperature for 2 hours. Finally, proteins were detected with the Odyssey® LI‐COR Imaging System (LI‐COR Biotechnology, USA).

### Enzyme-linked immunosorbent assay (ELISA)

ELISA was used to assess VEGFA protein level in cell culture medium. HEPM and HEK-293 cells were cultured in 6-well plates and transient transfected with *VEGFA* plasmids. 24 hours after transfected, fresh medium was changed. After further incubation for 24 hours, the supernatants of the cells were collected. Centrifuge the cell supernatants at 400 g to remove cell debris. Then, the concentration of VEGFA in the supernatants were detected using the Human VEGF ELISA Kit (Proteintech, China), following the user’s instruction, and calculated using ELISACalc according to the standard curve.

### Proliferation assay and iCELLigence real-time cell analysis system (RTCA)

The transfected HEPM and HEK-293 cell proliferation was measured by absorbance using a Cell Counting Kit-8 assay (CCK8, Beyotime, China) following the user’s instruction. HEPM cells were seeded in 96-well plates at approximately 5000 cells per well. After cells were transfected for 0, 24, 48, 72, 96 h with the wild-type/mutant *VEGFA* plasmids or control vector, 10 μl of CCK8 reagent were added per well. After incubating at 37°C in 5% CO_2_ for 1 h, the absorbance at 570 nm was determined by the microplate reader.

Fifteen thousand HEPM cells were seeded in each E-Plate L8 well (ACEA Biosciences, USA). 24 h after cell seeding, wild-type/mutant *VEGFA* plasmids, or control vector were transferred to HEPM cells. Then, the cells were monitored every 15 minutes by the RTCA iCELLigence instrument (ACEA Biosciences, USA).

### Wound-healing assay

HEPM and HEK-293 cells were cultured in 6-well plates. When the cells became confluent 24 hours after transfection, the monolayer was scratched by the 200 μl pipette tip to make a wound of about 750 μm in width. All the scratches were made using one pipette tip to ensure that the width of each scratch was approximately the same per well. The images taken at 0, 24, 48 hours after scratching were preserved.

### Cell cycle and apoptosis experiments

We used Cell Cycle Analysis kit (4A Biotech, China) to analyze cell cycle following the user’s instruction. 48 h after transfection with plasmids, HEK-293 and HEPM cells were washed three times with cold PBS, and fixed in 70% ethanol 12 h at 4°C. Wash again with cold PBS to remove ethanol. Under the dark condition of 37°C, cells were stained with 400 μl propidium iodide (PI)/RNase staining buffer for 30 min. Finally, flow cytometric analysis were performed on ACEA NovoCyte (ACEA Biosciences, USA). Cell cycle distribution was determined with NovoExpress software (ACEA Biosciences, USA). Cell apoptosis analysis was performed by using the Annexin V Alexa Fluor647/PI kit (4A Biotech, China). NovoExpress software (ACEA Biosciences, USA) was used to calculate apoptosis rate.

### RNA-seq

Total RNA in HEPM and HEK-293 T cells was extracted and its quantity and quality were evaluated by Nanodrop and 1% agarose gel electrophoresis. First, mRNA libraries were constructed from HEPM cells. High throughput sequencing was performed on Hiseq4000 platform and raw reads were obtained. After filtering the original data, checking the error rate of sequencing and checking the distribution of GC content, the clean reads was obtained for following analysis. After quality control, use HISAT2 software to align clean reads with GRCh38/hg38 human reference genome. Then, Then the difference of gene expression was analyzed by DESeq2 software. Gene ontology (GO) functional enrichment analysis and Kyoto Encyclopedia of Genes and Genomes (KEGG) pathway enrichment analysis of differential gene was carried out by clusterProfile software.

### RNA isolation and real-time PCR

Total RNA of cells was harvested using Ultrapure RNA Kit (Cwbiotech, China) after plasmids had been transfected into HEPM and HEK-293 cells for 24 h. The purity and concentration of total RNA were detected by the Nanodrop 8000 (Thermo scientific, USA). Residual DNA was removed from RNA and cDNA was generated by RNA reverse transcription using Super plus qPCR RT kit (Mei5bio, China). The relative mRNA levels were detected by real-time PCR with GAPDH as an internal control, using SYBRgreen (Mei5bio, China). The primer sequences are listed in Table 1.

**Table 1.** qPCR primer sequences

Gene	Forward primer sequence (5′–3′)	Reverse primer sequence (5′–3′)
GAPDH	TCAGCAATGCCTCCTGCAC	TCTGGGTGGCAGTGATGGC
Runx2	GATGACACTGCCACCTCTGAC	GGGATGAAATGCTTGGGAAC
ALP	TCAGAAGCTCAACACCAACG	TTGTACGTCTTGGAGAGGGC
OCN	TCACACTCCTCGCCCTATTG	CTCTTCACTACCTCGCTGCC
OSX	TCTCCCTCCCTCTCCCTTT	ATCATTAGCATAGCCTGAG
TGFβ1	CAGCAACAATTCCTGGCGATACC	GCGCTAAGGCGAAAGCCCTCAAT
BMP2	ACCCGCTGTCTTCTAGCGT	TTTCAGGCCGAACATGCTGAG

### Alkaline Phosphatase (ALP) activity analysis

MC3T3 cells were seeded in 24-well plates, and were cultured and differentiated in the osteogenic differentiation medium. 3 days after transfection with wild-type/mutant VEGFA plasmid or control vector, ALP activity analysis was carried out using the Alkaline Phosphatase Assay Kit (Beyotime, China) following the manufacturer’s protocols. Finally, OD values of 405 nm were detected using the microplate reader. The amount of alkaline phosphatase required per minute to hydrolyze para-nitrophenyl phosphate chromogenic substrate to produce 1 micromole of p-nitrophenol is defined as a unit of enzyme activity.

### Quantitative alizarin red s staining

After transfection of wild type/mutant VEGFA plasmid or control vector, MC3T3 cells cultured 7 days in the osteogenic differentiation medium. Briefly, removing the culture medium, the cells were washed twice with PBS and fixed in 4% paraformaldehyde for 10 min. Then, cells were stained using 0.2% alizarin red s (Solarbio, China) for 30 min to evaluate the mineralization and ossification. After dying, destain the cells for 30 min at room temperature by adding 10% (w/v) cetylpyridinium chloride (CPC), and the absorbance of extracted dye was measured at 562 nm.

### Statistical analysis

All graphs and data were shown as the mean ± standard deviations (SD). The two-sample Student’s *t*-test was used to calculate the significant differences between different groups. *P *< 0.05 was considered statistically significant.

## RESULT

### *Overexpression of* VEGFA *gene in cells*

In order to verify whether the *VEGFA* plasmids were transferred into the cells and expressed in the cells, total proteins were extracted 48 hours after transfection. The result of western blot assay shown that the expression of VEGFA proteins in both wild type and mutant *VEGFA* cells was significantly increased compared with the negative control (Figure 1a). However, the protein expression of mutant VEGFA and wild-type VEGFA was not significantly different.

### The stability of mutant VEGFA protein was lower than wild-type VEGFA

In order to find out the effect of the *VEGFA* mutation on VEGFA secretion, concentrations of VEGFA in the transfected HEK-293 and HEPM cell supernatants were measured by ELISA 48 hours after transfection (Figure 1b). ELISA results also proved that VEGFA was overexpressed in both two kinds of cells due to the existence of wild type/mutant *VEGFA* plasmids. The level of VEGFA in the supernatant of wild type cells was significantly higher than that of mutant cells. Combined with the western blot assay results, it is induced that the mutant VEGFA protein had a poorer stability in cell supernatants.

### The function of mutant VEGFA in promoting cell proliferation is weakened

**Figure 2.**
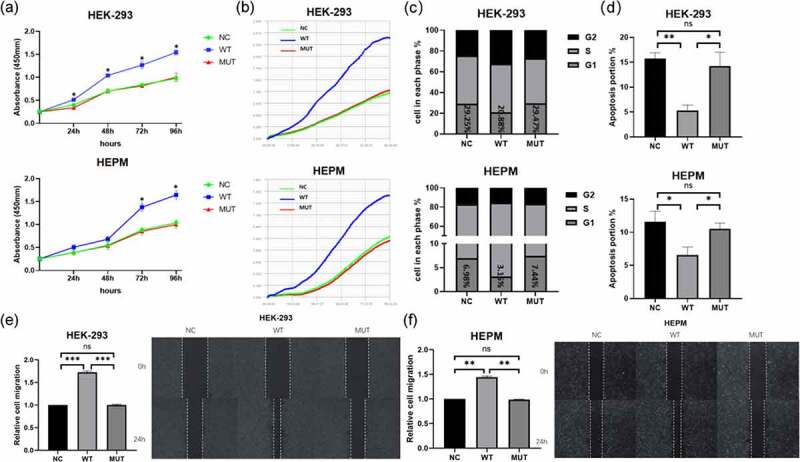
In vitro functional roles of wild-type/mutant *VEGFA* allele in HEPM and HEK-293 T. (a, b) Effect of *VEGFA* expression on cell proliferation. (a) HEPM and HEK-293 cells were transfected with wild-type VEGFA, mutant VEGFA or control vector (negative control), cell proliferation was detected every 24 h for 96 h by CCK-8 assay. (b) Cells were seeded in E-Plate L8 plates. After transfection with wild-type VEGFA, mutant VEGFA or control vector, cell proliferation was monitored every 15 minutes by the RTCA iCELLigence instrument. Cell proliferation in wild type group was significantly higher than in mutation group and negative control group. (c) Effects of different type of VEGFA on the cell cycle. Overexpression of wild-type VEGFA alleles decreased the number of cells stagnant G1 phase, but mutant VEGFA alleles did not have the same effect. (d) Effects of VEGFA expression on cell apoptosis. The apoptosis rate was lower in both two kinds of cells overexpressing mutant VEGFA alleles than in the corresponding wild-type VEGFA alleles group. (e, f) The wound healing assay was performed in HEPM (e) and HEK-293 (f) cells. Cells were treated with wild-type/mutant VEGFA plasmid or control vector after scratching. HEPM and HEK-293 T cells overexpressing wild-type VEGFA allele migrated 42% and 32% quicker than cells overexpressing mutant VEGFA allele, respectively. n = 3 for each group. *P < 0.05, **P < 0.01, ***P < 0.001. ns, no significance

To detect the impact of c.773 T > C *VEGFA* allele in the cells, HEK-293 and HEPM cells were transfected with vectors containing wild-type or mutant alleles and CCK8 assays, as well as iCELLigence Real‐Time Analysis system, was used to detect cell proliferation. The results of CCK-8 assays showed that the wild type VEGFA significantly increased cell proliferation (Figure 2a). However, there was no difference in the proliferation of the cells transfected with the mutant *VEGFA* alleles compared with the control groups.

We seeded HEK-293 and HEPM cells in the medium on the iCELLigence Real‐Time Analysis system and then treated cells with wild-type/mutant *VEGFA* plasmids or control vectors. Similar results of RTCA were found (Figure 2b). Flow cytometric analysis showed that overexpression of the mutant *VEGFA* alleles induced a significantly increase in the number of cells in G0/G1 phase and decrease in cells in S phase in cells compared to overexpressing wild-type *VEGFA* alleles (Figure 2c). The results of cell apoptosis are as the same (Figure 1d). These results meant that the mutant VEGFA protein lost its ability to promote cell proliferation.

### Mutation deprives VEGFA of its role in promoting cell migration

Wild type and mutant VEGFA proteins were overexpressed in HEPM and HEK‐293 cells. According to the result of western blot, the expression levels of wild type and mutant VEGFA protein are the same. When HEK-293 cells and HEPM cells became confluent, scratch wounds were made and wild-type and mutant VEGFA plasmids or control vectors were added to the cells. After 48 h, cells that had been treated with the wild type *VEGFA* plasmids migrated quicker than cells that had been treated with the mutant plasmids or negative controls (Figure 2e, Figure 2f). Therefore, mutant VEGFA could not promote cell migration.

### Identification of differentially expressed genes (DEG)

**Figure 3.**
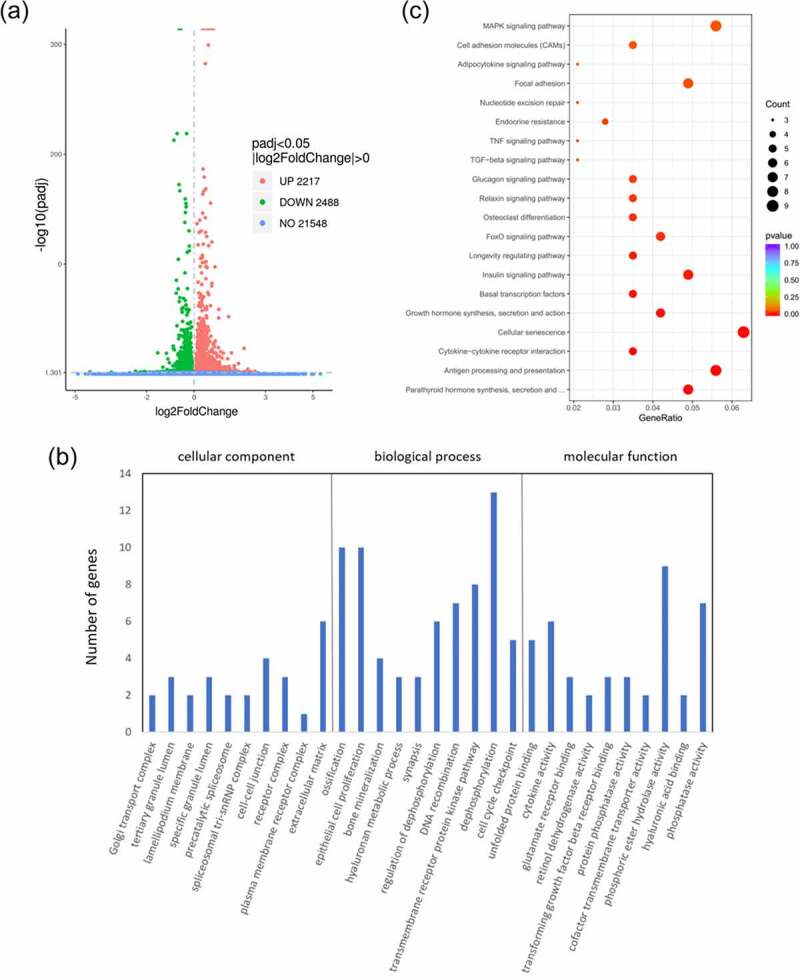
Differential analysis, GO analysis and KEGG pathway analysis of differential gene expression between cells overexpressing wild-type VEGFA and mutant VEGFA. (a) Volcano Plot. Compared with the wild-type VEGFA group, mutant VEGFA triggered 4705 DEGs, containing 2488 downregulated genes (green dot) and 2217 upregulated genes (red dot). (b) GO analysis histogram. 30 enrichment GO entries were shown, according to biological processes, cellular components, and molecular functions. (c) KEGG pathway analysis scatter diagram. 20 significant enriched pathways, including MAPK signaling pathway, TNF signaling pathway, TGFβ signaling pathway and osteoclast differentiation, were shown

From difference analysis, we obtained the genome-wide transcription of HEPM cells that were transfected with wild-type/mutant *VEGFA* plasmids or control vectors. 4705 DEGs were found between the wild type VEGFA group and the mutant VEGFA group, of which 2488 genes were downregulated and 2217 genes were upregulated (Figure 3a).

### GO analysis and pathway analysis

GO analysis of DEGs induced by wild-type VEGFA and mutant VEGFA was performed. GO is a comprehensive gene function database, which can be divided into three parts: biological process, cellular component and molecular function. We used GO analysis to assess the relationship between DEGs and their main functions. As a result, we drew the GO statistical histogram of DEGs based on biological processes, cellular components, and molecular functions (Figure 3b), showing 30 enrichment GO entries. Moreover, we also performed the pathway analysis based on the KEGG database (Figure 3c). KEGG pathway enrichments took p < 0.05 as the threshold of significant enrichments. Twenty significant enriched pathways were shown in Figure 3c, including TGFβ signaling pathway, TNF signaling pathway, osteoclast differentiation and MAPK signaling pathway.

### qPCR analysis of the DEGs

**Figure 4.**
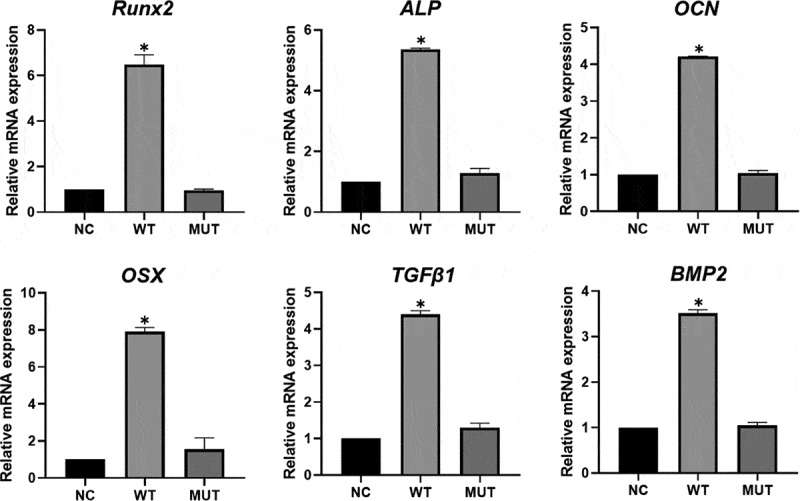
Expression of osteogenesis-related genes by qPCR analysis. The expression of *Runx2, OSX, BMP2, ALP, OCN* and *TGFβ1* was significantly higher in wild type VEGFA group, and had no significant in mutant VEGFA group, compared with the control group. Most importantly, the expression of these genes in the mutant VEGFA group was significantly lower than in the wild type group

We selected six osteogenesis-related genes, *ALP, Osterix* (*OSX), Osteocalcin* (*OCN), BMP2, Runx2, TGFβ1*, which were significantly downregulated in mutant VEGFA group than wild type group in GO analysis and pathway analysis, to perform qPCR (Figure 4). The results of qPCR showed the expressions of these genes were significantly decreased in HEPM cells overexpressed mutant VEGFA compared with the cells overexpressed wild type VEGFA. Fortunately, the results of RNA-seq are consistent with that of qPCR verification.

### The mutation in VEGFA partly inhibited osteoblast differentiation

MC3T3 is commonly used in the study of osteoblast differentiation. ALP is a membrane glycoprotein of osteoblasts and is widely used as a marker of osteogenesis [22]. Alizarin red can detect and combine with calcium salts in tissues and cells to form complexes, which is one of the markers of osteoblasts proliferation and differentiation and osteogenic potential of bone tissue.

**Figure 5.**
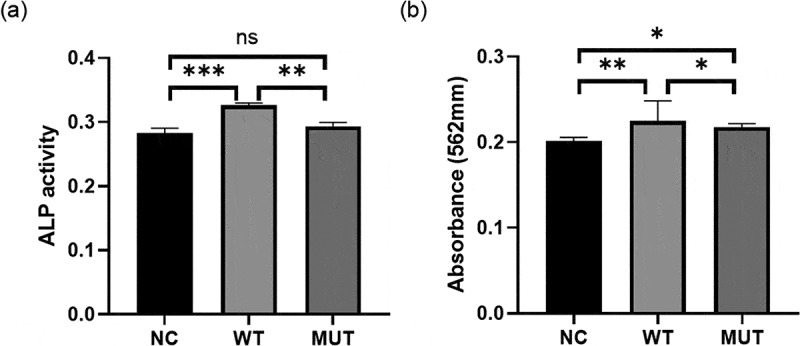
VEGFA promotes osteogenesis, while the mutation of VEGFA reduced the osteogenic effect. (a) ALP activity analysis. MC3T3 cells transfected with wild-type/mutant VEGFA plasmids or control vectors were cultured in osteogenic medium for 3 days and stained by ALP content. (b) Alizarin red stain. The transfected MC3T3 cells cultured for 7 days were stained by alizarin red. n = 3 for each group. *P < 0.05, **P < 0.01, ***P < 0.001. ns, no significance

To investigate the effect of the mutation on VEGFA in osteogenesis, MC3T3 cells were transfected with wild-type/mutant *VEGFA* plasmid to overexpress VEGFA protein, accompanied with cells transfected with empty vector as negative controls. We found higher ALP activity in cells with wild-type VEGFA than in mutant VEGFA and control cells, while there is no statistical difference of ALP activity between mutant VEGFA and control cells (Figure 5a). The degree of mineralization of cells overexpressing wild-type VEGFA were also higher than the other two groups, which was determined via alizarin red s staining (Figure 5b). The results of ALP activity analysis and quantitative alizarin red s staining were consistent, indicating that the mutation of VEGFA decreased osteogenesis.

## DISCUSSION

By the fourth week of the embryonic period, the five protuberances, including the frontonasal prominence, two maxillary prominences, and two mandibular prominences, was formed around the primitive oral cavity. The maxillofacial region developed from these five protuberances. At the end of the sixth week, the primary palate and upper lip were formed after medial nasal processes had merged with the maxillary prominences. The secondary palate developed when the bilateral maxillary prominences formed outgrowths at the sixth week in embryonic period. The palatal plates first grew down vertically along the side of the tongue, then elevated to a horizontal position above the tongue, touched each other and began to fusion [13]. Human palate developed from the primary palate and the secondary palate. The primary palate only formed a small part of the hard palate, which mainly developed by the secondary palate. The secondary palate was also the primordium of the soft palate [23]. Therefore, palatogenesis is a process with multiple steps, including the growth of palatal shelf, elevation, fusion at the midline and the disappearance of the medial edge epithelium (MEE) [24,25]. Abnormalities at any stage of palate shelves formation will lead to cleft palate, such as failure of palatal shelf formation, fusion of the palatal shelf with the tongue or mandible, palatal shelf elevation failure, failure of fusion after elevation and persistence of medial edge epithelium [23].

Cleft palate (CL/P) has been divided into isolated CL/P and cleft lip with or without CL/P [26]. About 70% of CL/P cases and 50% of isolated CL/P cases are considered to be nonsyndromic [27]. As this defect occurs in the early stage of embryonic development, has a multifactorial etiology affecting by both genetic factors and environmental factors and has a modest recurrence rate, it is hard to determine the exact etiology. However, it is essential to understand the biological processes of maxillofacial development and the mechanism of interaction between genetic risks and environmental conditions, and to improve clinical treatment for known causes. The application of epidemiology, candidate genes, GWAS and animal models analysis have led to a deeper insight into the etiology of NSCL/P The application of epidemiology, genetic tools and animal model analysis makes reseachers have a better understanding of the cause of NSCL/P [13]. In our previous WES study in a NSCL/P pedigree, we identified A novel *VEGFA* mutation (NM_001025366.2 c.773 T > C p.Val258Ala) as a candidate for causing NSCL/P in this case [16]. According to the three-dimensional structure of the VEGFA protein, this VEGFA variant may disrupt the hydrophobic interaction of VEGFA, thus reducing the stability of VEGFA homodimer and the affinity to its receptors.

The appearance of cranial neural crest (CNC) cells is one of the crucial characteristics of craniofacial development. The survival, migration, proliferation, apoptosis and ultimate outcome of the CNC cells are closely related to the regulation of craniofacial development. CNC cells make up most of the palatal mesenchyme. Genetic disorder, environmental abnormalities, or both of them influence the outcome of CNC cells, and, severely, lead to deformities in craniofacial region [28,29].Moreover, *VEGFA* is required by CNC cells during the process of embryonic development.

VEGFA is a secretory homologous dimer protein. There are at least six different transcripts of human *VEGFA* gene due to different splicing, including VEGFA_121_, VEGFA_145_, VEGFA_165_, VEGFA_183_, VEGFA_189_ and VEGFA_206_ [30,31]. In tissues, VEGFA_165_ is the most frequently expressed isoform among these isoforms. VEGFA binds to VEGFA receptors and regulates proliferation and migration of vascular endothelial cells, and vascular sprout, which contribute to the development of vascular [32].

According to previous studies, the cell proliferation, migration and apoptosis of CNC-derived HEPM cells play a significant role in palatogenesis [33]. Abnormalities in these processes may result in the occurrence of cleft palate [34]. Our vitro experiments showed that the wild type *VEGFA* allele can be promoted the HEK-293 and HEPM cells proliferation and lead to a significant inhibition in G0/G1 phase cells, while the number of cells in S phase augment significantly. However, the mutant *VEGFA* allele did not have the same effects, indicating that the mutant allele restrains the proliferation of both two kinds of cells. Therefore, we used wound-healing assay to evaluate migration of both two kinds of cells with wild-type/mutant *VEGFA* allele. We observed that the migration rate of cells treated with mutant VEGFA plasmid was slower than that of cells treated with wild type plasmid. This result indicated that the wild-type *VEGFA* allele but not mutant *VEGFA* allele promoted the migration of the cells. Furthermore, the outcomes of apoptosis experiment showed that the mutant *VEGFA* protein cannot restrain apoptosis as the wild-type *VEGFA* protein dose.

VEGFA is also an important growth factor necessary for bone development. The maxillary and palatal bone are formed by CNC-derived mesenchymal cells, which are the main cell types of maxilla and palate, through intramembranous osteogenesis [35]. Previous animal model studies have also shown that *VEGFA* is related to osteogenesis. *VEGFA^120/120^* mice, which only express *VEGFA^120/120^* and fail to express another *VEGFA* isoforms, had reduced ossification and vascularization of long bone, which is related to the decreased differentiation of osteoblasts and endothelial cells [36]. *VEGFA*CKO in the CNC can reduce cell proliferation, resulting in the damage of the elongation and elevation of the palate shelf and cleft palate in mice [10].

We found that in RNA-seq, compared with wild type *VEGFA* alleles, the expression of osteoblast fate-related genes in HEPM cells treated with mutant *VEGFA* alleles was significantly downregulated. The relatively low expression of *Runx2* and *ALP* in cells with mutant *VEGFA* allele indicates that mesenchymal cells are less transform to osteoblast. The decrease of *OSX* and *OCN* in cells transfected with mutant *VEGFA* allele plasmid indicated that, may be due to the less involvement of osteoblasts, the reduced expression of determinants in the late stage of bone development. Moreover, MC3T3 cells were transfected with wild-type and mutant *VEGFA* plasmids, respectively, and ALP activity and quantitative alizarin red s staining were used to detected osteogenic differentiation and mineralization. The RNA-seq results suggested the stimulating effect of wild-type VEGFA on osteoblast differentiation was higher than that of mutant type, indicating that the mutation has a negative influence on osteogenesis. Oliver et al [37] also revealed that the tissue engineering scaffold of osteogenic growth factor is involved in regulating the molecular signal network related to driving the occurrence of CL/P, providing a new way for the CL/P surgical correction operation site to stimulate bone formation.

In vitro, compared with the wild-type *VEGFA* allele, mRNA expression of *TGFβ1* and *BMP2* were reduced in HEPM cells with mutant *VEGFA* allele. *TGFβ, BMP* and *VEGFA* signaling are strongly associated with the development of blood vessels and bones. According to previous study, in mouse osteoblast MC3T3, *TGFβ1* can upregulate the transcription level of VEGFA. Conversely, *TGFβ1* treatment also significantly increased the secreted level of VEGFA. These results suggested that VEGFA may involve in the osteogenesis of *TGFβ1* [38,39]. The differentiation and ossification of osteoblasts in VEGFACKO mice were decreased, and the growth of maxilla and palate bone was insufficient. Moreover, the inhibition of differentiation and ossification of osteoblasts can be rescued by adding BMP2 and the addition of VEGFA can enhance this effect in vitro [10].

## CONCLUSION

Our study found that the mutation in *VEGFA* (NM_001025366.2 c.773 T > C p.Val258Ala) deprives the function of promoting cell proliferation and migration and inhibiting apoptosis, which were belonging to VEGFA. Moreover, the stability of mutant VEGFA may be reduced. The mutation also affects the expression of downstream osteogenic genes, thus affecting osteogenesis. These findings suggested that *VEGFA* mutation (c.773 T > C) may affect the palatal development and may well be the cause of NSCL/P in the family we recruited, which further proved the important role *VEGFA* gene played in the etiology of NSCL/P. In addition, understanding the processes of angiogenesis and osteogenesis, and the relationship between them may help surgeons to solve the potential bone growth and wound healing problems in patients with CL/P after repair.

## Supplementary Material

Supplemental Material

## Data Availability

Data and materials in this article are available.

## Highlights

1. The *VEGFA* mutation (c.773T>C) inhibits cell proliferation and migration and promotes apoptosis in HEPM cells;
2. The *VEGFA* mutation has a negative influence on osteogenesis of HEPM cells;
3. The *VEGFA* mutation may impact the development of the palate.
