# Supplementary material for: Functional identification of a rare vascular endothelial growth factor a (VEGFA) variant associating with the nonsyndromic cleft lip with/without cleft palate
Source: Bioengineered. 2021 May 5;12(1):1471–83. doi: 10.1080/21655979.2021.1912547 (PMC8806239; doi:10.1080/21655979.2021.1912547)
Supplement: Supplemental Material [file KBIE_A_1912547_SM0001.rtf]

Supplementary figure 1. The structure of VEGFA plasmid. The mutant plasmid contains a missense mutation (red arrow).
